# Independent effect of physical activity and resting heart rate on the incidence of atrial fibrillation in the general population

**DOI:** 10.1038/s41598-019-47748-7

**Published:** 2019-08-02

**Authors:** Yeon Woo Choi, Minsu Park, Young-Hyo Lim, Jisun Myung, Byung Sik Kim, Yonggu Lee, Jeong-Hun Shin, Hwan-Cheol Park, Jinho Shin, Chun Ki Kim, Jin-Kyu Park

**Affiliations:** 1 Division of Cardiology, Department of Internal Medicine, Hanyang University Medical Center, Seoul, Republic of Korea; 2 Statistics and Data Center, Samsung Biomedical Research Institute, Samsung Medical Center, Seoul, Republic of Korea; 3 Department of Preventive Medicine, School of Medicine, Kyunghee University, Seoul, Republic of Korea; 4 Division of Cardiology, Department of Internal Medicine, Hanyang University Guri Hospital, Guri City, Gyounggi-do Republic of Korea; 5 Department of Nuclear Medicine, Hanyang University Medical Center, Seoul, Republic of Korea

**Keywords:** Risk factors, Atrial fibrillation, Epidemiology

## Abstract

While physical activity (PA) may influence resting heart rate (RHR), and a low RHR may be a risk factor for atrial fibrillation (AF), controversy exists regarding the association between PA and development of AF. Using data from a Korean, prospective population cohort, we investigated the independent effect of PA and RHR on the incidence of AF in the general population. A total of 8,811 participants aged 40–69 years were analyzed. Total PA assessed based on questionnaires was divided into quartiles, with the lowest to the highest being Q1, Q2, Q3, and Q4. During a median follow-up of 139 months, AF developed in 167 participants (1.9%). Q3 of total PA was associated with a significantly lower risk of AF than Q1 even after adjusting for RHR as a covariate, but Q4 was not. The risk of AF was higher in participants with RHR < 60 bpm than in those with RHR 70–85 bpm, and the significance persisted after adjusting for PA as a covariate. This study showed that a moderate amount of total PA was associated with a lower risk of incident AF independent of RHR and that low RHR was an independent risk factor for AF in the general Korean population.

## Introduction

Atrial fibrillation (AF) is the most common arrhythmia and is an independent risk factor for cardiovascular events including heart failure, and ischemic stroke^1^. Risk factors for the development of AF include hypertension, diabetes, obesity, smoking, ischemic heart disease, and heart failure.

Association between physical activity (PA) and AF has also been studied, and moderate PA is reported to be associated with a reduced risk of AF, while higher PA is not^2,3^. On the other hand, a meta-analysis showed that the trend toward lower risk of AF in subjects practicing PA is non-significant and that the extreme heterogeneity of published studies does not allow conclusive results^4^.

There are other issues related to PA. While previous studies divided PA into the light, moderate, and vigorous activities, none of them quantified the effect of total daily life PA. In addition, it is still not completely clear whether the PA and resting heart rate (RHR) have an independent effect on the development of AF. Endurance athletes are more likely to develop AF than non-athletes, with sinus bradycardia and increased vagal tone as possible pathophysiological mechanisms^5^. Therefore, PA and RHR may presumably also influence each other in the general population, and a relation between RHR and incidence of AF has indeed been suggested^6,7^. However, there are limited data about the interplay between PA and RHR in the development of AF^8^. Further, there have been no large-scale studies analyzing the effect of PA and RHR on the incidence of AF in an Asian population.

Our study aimed to assess (1) whether the total PA and RHR have an independent effect on incident AF and (2) whether the amount of total daily activity has any influence on the risk of AF in the general population based on a prospective population study carried out in South Korea.

## Methods

### Study population

The Ansung-Ansan cohort is a prospective study population that consists of South Koreans aged 40–69 years residing in two cities, Ansan and Ansung. This study was started in 2001 and embedded within the Korean Genome and Epidemiology Study (KoGES), a population-based cohort study, to assess the incidence and risk factors for various chronic disorders including cardiovascular diseases.

The baseline survey, conducted from 2001 to 2002, included 10,030 adults. Following the baseline visit, these adults were examined every two years with the longest follow-up duration being 12 years. At each of the baseline and biennial follow-up visits, data on lifestyle characteristics, cardiovascular profiles, medical history, and disease incidence were collected. All medical tests and the interview-based surveys were conducted by health professionals who were trained to follow a standardized protocol, and the same instruments were used to collect the data. More detailed information regarding the enrollment of cohort members and study procedures is available in a previous report^9^.

### Analytical sample

Among the 10,030 subjects, we analyzed 8,811 (4,235 males and 4,576 females), excluding participants whose heart rate measurement was not available (n = 15), those with AF at baseline (n = 40), those with no follow-up information (n = 965), and those with missing information on covariates (n = 199). All of the study protocol were approved by the Ethics Committee of KoGES at the Korean National Institute of Health and adhered to the principles of the Declaration of Helsinki. Informed consent was obtained from all subjects. This study was approved by the Institutional Review Board of Hanyang University Medical Center (IRB file No. HYUH 2017-12-033).

### Physical activity measurement

PA was assessed using a questionnaire to quantify activities in occupational or leisure time domains and was categorized into 5 levels: steady state, sitting state, and light, moderate, and high activity. A corresponding metabolic equivalent (MET) was assigned for each level as follows: (1) steady state (MET 0) - lying down; (2) sitting state (MET 1.5) - typing, driving, office work, playing a musical instrument, sewing, studying, ironing, writing, and cooking; (3) light activity (MET 3) - walking, cleaning, laundry, child care, bathing, and exercise as entertainment (bicycle, table tennis); (4) moderate activity (MET 5) - fast walking, woodworking, lawn mowing, snow removal, and regular exercise (badminton, swimming, tennis); and (5) high activity (MET 7) - athletics, climbing, running, woodcutting, agriculture, forestry, and mining. The questionnaire included questions about whether participants performed each of the above 5 levels of PA ≥ 1 h/day or < 1 h/day. The total PA in MET-h/day was based on the PA intensity and the time commitment for each PA.

### Measurement of resting heart rate and other covariates

RHR was recorded by measuring the pulse for 60 s. Covariates including baseline age, sex, urban or rural residence, education level, past medical history, smoking status, and alcohol consumption were evaluated based on self-reports. Blood pressure (BP) was measured by well-trained nurses after a 5-min rest. Hypertension was defined as systolic BP > 140 mmHg, diastolic BP > 90 mmHg, or the use of antihypertensive medication. We defined a participant as diabetic if any of the following criteria were met: current use of an oral hypoglycemic agent or insulin or a serum fasting glucose ≥126 mg/dl. A participant was categorized as having hyperlipidemia if any of the following criteria were met: current use of lipid-lowering medication, serum total cholesterol ≥ 240 mg/dl, or serum triglyceride ≥200 mg/dl.

### Follow-up and detection of AF

Biennial follow-up examinations and electrocardiograms (ECGs) were performed until 2014. A resting supine 12-lead ECG (MAC 5000^®^; GE Marquette Inc., Milwaukee, WI, USA) was obtained during quiet respiration. The ECG was recorded at 25 mm/s with 0.1 mV/mm standardization and was interpreted by a cardiologist to confirm the diagnosis and classified according to the Minnesota code. AF was defined as a composite of AF or atrial flutter in the standard 12-lead ECG.

AF at baseline was identified by ECG performed during the baseline visit and/or the self-reported history of physician-determined diagnosis made prior to the baseline visit. Similarly, AF newly developed after the baseline visit was also identified by biennially performed ECGs and/or the self-reported history of physician-determined diagnosis that was made between each biennial visit. If AF was identified by both ECGs and the history in the same subject, the earlier identification date was considered the time of AF development.

### Statistical analyses

Continuous variables were presented as a median and interquartile range; Kruskal-Wallis test and Dunn’s multiple comparison test (post-hoc analysis), comparing all groups to controls were used to compare continuous variables among groups. Categorical variables were presented as numbers and percentages. The chi-squared test was used for categorical variables. A Cox proportional hazards regression model was used to assess the association between PA, RHR, and incidence of AF. The collected total PA (MET-h/day) was divided into quartiles, with the lowest and the highest being Q1 and Q4, respectively. Q1 was used as the reference. RHR was categorized into 5 groups (<50, 50–59, 60–70, 70–85, and >85 bpm). The RHR range of 70–85 bpm was used as a reference. The covariates were age, sex, residence, education, body mass index (BMI), comorbidity, smoking status, and alcohol consumption in models 1 and 2. In model 3, we added RHR or PA to evaluate the independent association with the incidence of AF. All statistical analyses were performed using R-3.4.0 software. A p < 0.05 was considered significant.

## Results

### Baseline demographic and health-related characteristics

The baseline characteristics according to the PA category are listed in Tables 1 and 2. A total of 8,811 participants (48.1% male) were followed up for a median period of 139 (97, 141) months. The median age was 50 (44, 60) years. There were several significant differences between participants in the lowest quartile (Q1) and the highest quartile (Q4): The median age of participants was higher, there were more male participants, the % of urban residents (=urban residents/(urban + rural residents) was lower, and the overall education level of participants was lower in Q4 than in Q1 (Tables 1 and 2). There was a significant difference in the distribution of occupations. The large majority (62.7%) of participants engaged in agriculture belonged in Q4. Conversely, the smallest proportion of participants with all other occupations belonged in Q4 (Table 1).

**Table 1:** Demographic characteristics according to quartiles of physical activity.

	Physical activity (METs/day)					
	Total	Q1	Q2	Q3	Q4	p-value
	19.5 (11.3, 35.6)	<11.3	11.3–19.4	19.5–35.5	≥35.6	
	n = 8,811	n = 2,230	n = 2,047	n = 2,251	n = 2,283	
Age (years)	50 (44, 60)	49 (44, 59)	48 (43, 57)^†^	49 (44, 58)	56 (48, 63)^†^	<0.001
Sex						<0.001
Male	4,235	1,072 (25.3%)	920 (21.7%)	1,068 (25.2%)	1,175 (27.8%)	
Female	4,576	1,158 (25.3%)	1,127 (24.6%)	1,183 (25.9%)	1,108 (24.2%)	
Residence						<0.001
Urban	4,317	1,229 (28.5%)	1,468 (34.0%)	1,378 (31.9%)	242 (5.6%)	
Rural	4,494	1,001 (22.3%)	579 (12.9%)	873 (19.4%)	2,041 (45.4%)	
Education						<0.001
≤Elementary school	2,958	673 (22.8%)	475 (16.0%)	599 (20.3%)	1,211 (40.9%)	
Middle/high school	4,666	1,199 (25.7%)	1,182 (25.3%)	1,314 (28.2%)	971 (20.8%)	
College/university	1,187	358 (30.2%)	390 (32.9%)	338 (28.5%)	101 (8.4%)	
Follow-up duration (months)	139 (97, 141)	139 (95, 141)	139 (97, 142)^†^	139 (108, 141)	139 (98, 142)^†^	0.002
Occupation						<0.001
Housewife	2,453	728 (29.7%)	711 (29.0%)	742 (30.2%)	272 (11.1%)	
Office work	391	123 (31.5%)	151 (38.6%)	98 (25.1%)	19 (4.8%)	
Agriculture	2,486	343 (13.8%)	175 (7.0%)	410 (16.5%)	1,558 (62.7%)	
Self-employment	1,250	349 (27.9%)	403 (32.2%)	381 (30.5%)	117 (9.4%)	
Sales	110	29 (26.4%)	42 (38.2%)	28 (25.4%)	11 (10.0%)	
Factory production	470	114 (24.3%)	125 (26.6%)	138 (29.4%)	93 (19.7%)	
Professional work	357	106 (29.7%)	101 (28.3%)	109 (30.5%)	41 (11.5%)	
Others	1,258	415 (33.0%)	336 (26.7%)	343 (27.3%)	164 (13.0%)	

Data shown with percentages in parentheses represent numbers of participants, and data shown with two numbers in parentheses represent median values with interquartile ranges in parentheses.

^†^p < 0.01 vs. Q1. Kruskal-Wallis test (Dunn’s post hoc).

**Table 2:** Baseline health-related characteristics according to quartiles of physical activity.

	Physical activity (METs/day)					
	Total	Q1	Q2	Q3	Q4	p-value
	19.5 (11.3, 35.6)	<11.3	11.3–19.4	19.5–35.5	≥35.6	
	n = 8,811	n = 2,230	n = 2,047	n = 2,251	n = 2,283	
Body mass index (kg/m^2^)	24.5 (22.5, 26.5)	24.5 (22.5, 26.7)	24.7 (22.8, 26.8)	24.5 (22.7, 26.4)	24.2 (22.0, 26.2)^†^	<0.001
Hypertension	1,351 (15.3%)	361 (16.2%)	309 (15.1%)	335 (14.9%)	346 (15.2%)	0.625
Diabetes mellitus	579 (6.6%)	171 (7.7%)	114 (5.6%)	143 (6.4%)	151 (6.6%)	0.048
Hyperlipidemia	215 (2.4%)	58 (2.6%)	70 (3.4%)	56 (2.5%)	31 (1.4%)	<0.001
Congestive heart failure	21 (0.2%)	5 (0.2%)	1 (<0.1%)	4 (0.2%)	11 (0.5%)	0.027
Coronary artery disease	67 (0.8%)	17 (0.8%)	20 (1%)	19 (0.8%)	11 (0.5%)	0.280
Previous myocardial infarction	84 (1%)	27 (1.2%)	11 (0.5%)	21 (0.9%)	25 (1.1%)	0.121
Peripheral artery disease	30 (0.3%)	8 (0.4%)	7 (0.3%)	6 (0.3%)	9 (0.4%)	0.901
Cerebrovascular disease	98 (1.1%)	32 (1.4%)	18 (0.9%)	29 (1.3%)	19 (0.8%)	0.146
Chronic obstructive pulmonary disease	59 (0.7%)	19 (0.9%)	11 (0.5%)	10 (0.4%)	19 (0.8%)	0.233
Asthma	193 (2.2%)	55 (2.5%)	42 (2.1%)	44 (2%)	52 (2.3%)	0.650
Thyroid disease	264 (3.0%)	64 (2.9%)	69 (3.4%)	82 (3.6%)	49 (2.1%)	0.018
Smoking habit						<0.001
Never	5,183 (58.8%)	1,294 (58%)	1,271 (62.1%)	1,337 (59.4%)	1,281 (56.1%)	
Former	1,384 (15.7%)	328 (14.7%)	315 (15.4%)	387 (17.2%)	354 (15.5%)	
Current	2,244 (25.5%)	608 (27.3%)	461 (22.5%)	527 (23.4%)	648 (28.4%)	
Alcohol						0.186
Never	4,045 (45.9%)	1,052 (47.2%)	932 (45.5%)	1,007 (44.7%)	1,054 (46.2%)	
Ex-	563 (6.4%)	149 (6.7%)	109 (5.3%)	152 (6.8%)	153 (6.7%)	
Current	4,203 (47.7%)	1,029 (46.1%)	1,006 (49.1%)	1,092 (48.5%)	1,076 (47.1%)	
Antihypertensive medication	1,025 (11.6%)	272 (12.2%)	237 (11.6%)	257 (11.4%)	259 (11.3%)	0.581
**Levels of physical activities**						
Steady ≥1 h/day	3,139 (36%)	846 (39.5%)	758 (37%)	865 (38.5%)	670 (29.4%)	<0.001
Sitting ≥1 h/day	6,736 (77.4%)	1,540 (72.7%)	1,714 (83.7%)	1,944 (86.4%)	1,538 (67.4%)	<0.001
Light ≥1 h/day	6,049 (69.7%)	842 (40%)	1,737 (84.9%)	1,924 (85.5%)	1,546 (67.7%)	<0.001
Moderate ≥1 h/day	2,120 (24.6%)	84 (4.1%)	249 (12.2%)	1,022 (45.4%)	765 (33.5%)	<0.001
High ≥1 h/day	2,834 (32.7%)	103 (4.9%)	121 (5.9%)	546 (24.3%)	2,064 (90.4%)	<0.001
Resting heart rate (bpm)	62.7 (59.3, 68)	63.3 (60, 68.3)	62.7 (59.3, 67.3)^†^	62.0 (58.7, 67)^†^	63.3 (60, 68)	<0.001
<50	137 (1.6%)	38 (1.7%)	33 (1.6%)	43 (1.9%)	23 (1%)	<0.001
50–59	1,872 (21.2%)	433 (19.4%)	486 (23.7%)	565 (25.1%)	388 (17%)	
60–69	5,119 (58.1%)	1,275 (57.2%)	1,181 (57.7%)	1,266 (56.2%)	1,397 (61.2%)	
70–85	1,590 (18%)	456 (20.4%)	335 (16.4%)	358 (15.9%)	441 (19.3%)	
>85	93 (1.1%)	28 (1.3%)	12 (0.6%)	19 (0.8%)	34 (1.5%)	

Data shown with percentages in parentheses represent numbers of participants, and data shown with two numbers in parentheses represent median values with interquartile ranges in parentheses.

^†^p < 0.01 vs. Q1. Kruskal-Wallis test (Dunn’s post hoc).

BMI was significantly lower in Q4 than in Q1. However, the differences in age and BMI between Q3 and Q1 were not significant. The frequencies of hyperlipidemia and thyroid disease were lower among participants in Q4 than those in the other three quartiles. There were more current or former smokers in Q4 than in other quartiles but no significant differences in alcohol consumption among four quartiles (Table 2).

### Physical activity and resting heart rate

Only the total PA measured at baseline was analyzed for the following reasons: 1) The total number of follow-up visits varied among participants; therefore, using the baseline data that were available in every participant would likely provide the most generalizable results. Further, including all data from varying numbers of follow-up visits could complicate the analysis, presentation, and interpretation of data. 2) After the baseline visit, there was some modification in the questionnaire for assessing PA for reasons unrelated to the present study; therefore, METs/day could not be calculated throughout all visits in a consistent manner.

For each component of PA according to total PA quartiles (Table 2), light activity ≥1 h/day was performed by more than half of participants in all quartiles except Q1. The percentage of participants performing moderate activity ≥1 h/day was highest (45.4%) in Q3. While the percentage of participants in Q4 performing moderate PA ≥ 1 h/day (33.5%) was not as high, 90.4% of participants in Q4 performed high PA ≥ 1 h/day (vs. 24.3% in Q3). More detailed components of PA in each of the four quartiles of total PA are shown in Supplementary Fig. 1.

Only baseline RHR data was analyzed for the same reason as “Reason 1” mentioned in the first paragraph of this section and also because initial rankings of 5 RHR groups determined based on median values of baseline RHR remained identical throughout all follow-up visits (Supplementary Fig. 2).

Comparing the baseline RHR among quartiles (Table 2), Q3 included the highest percentage of subjects with RHR < 60 bpm (27%), and the RHR was significantly lower in Q3 than in Q1 (62.0 [58.7, 67] bpm, p < 0.001). On the other hand, Q4 had the lowest percentage with RHR < 60 bpm (18%).

### Physical activity, resting heart rate and incident AF

**Figure 1:**
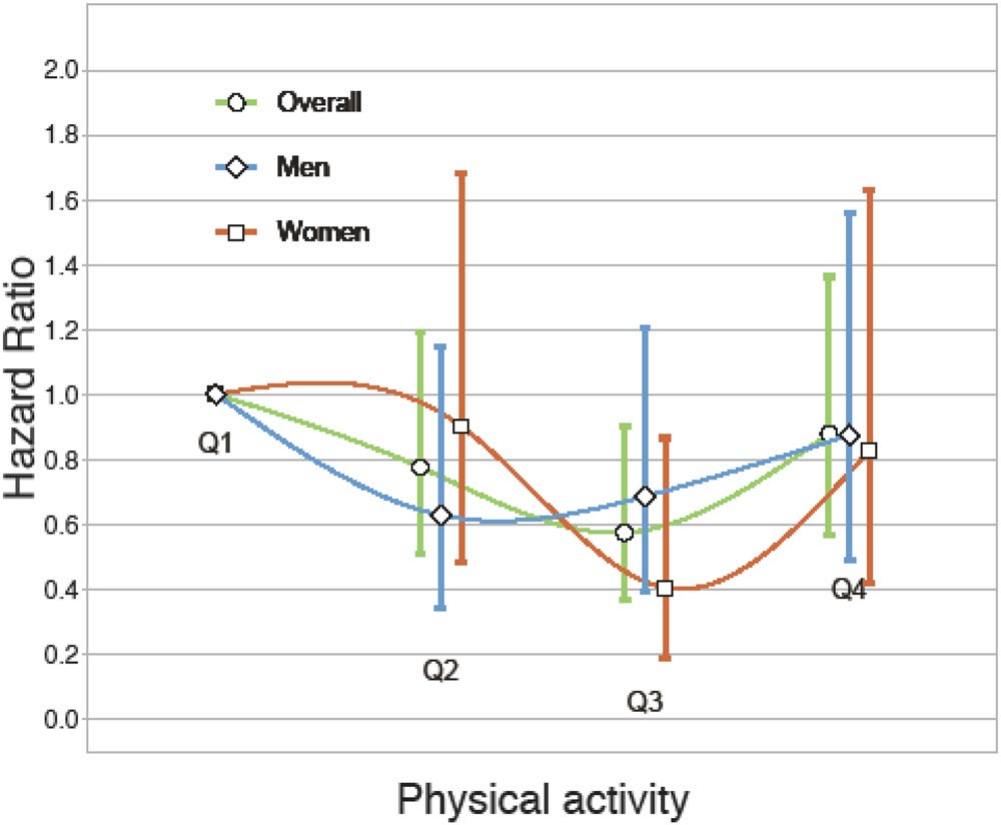
Risk of atrial fibrillation according to the level of physical activity.

There was a gradual decrease in the incidence rate and risk of AF from Q1 to Q3 (Table 3); Q3 had the lowest incidence rate of AF (1.42/1,000 person-years) and a significantly lower risk of AF, after adjusting for covariates, than Q1 and Q4. This trend remained consistent after adjusting for RHR (hazard ratio [HR] 0.58, 95% confidence interval [CI] 0.37–0.90, p = 0.016). On the other hand, Q4 had a high incidence rate of AF (2.12/1,000 person-years); the risk of AF was not even lower than in Q1 (HR 0.88, 95% CI 0.57–1.36). These associations between PA and incident AF were similar in both male and female subjects, but were more significant in females (Fig. 1).

**Table 3:** Hazard ratio for incident atrial fibrillation according to quartiles of total physical activity.

		Physical activity (METs/day)			
		Q1	Q2	Q3	Q4
No. with AF		51 (2.3%)	38 (1.9%)	31 (1.4%)	47 (2.1%)
Incidence rate		2.39	1.91	1.42	2.12
Crude	HR (95% CI)	1	0.79 (0.52–1.20)	0.59 (0.38–0.92)	0.87 (0.59–1.29)
	p-value		0.263	0.020	0.489
Model 1	HR (95% CI)	1	0.78 (0.51–1.20)	0.58 (0.37–0.91)	0.84 (0.54–1.30)
	p-value		0.255	0.017	0.425
Model 2	HR (95% CI)	1	0.79 (0.52–1.21)	0.59 (0.38–0.92)	0.90 (0.58–1.39)
	p-value		0.273	0.021	0.622
Model 3	HR (95% CI)	1	0.78 (0.51–1.19)	0.58 (0.37–0.90)	0.88 (0.57–1.36)
	p-value		0.246	0.016	0.564

Model 1: adjusted for age, sex, residence, education.

Model 2: Model 1 + BMI, comorbidity, alcohol and smoking.

Model 3: Model 2 + RHR.

AF, atrial fibrillation; Incidence rate, per 1,000 person-years; HR, hazard ratio; CI, confidence interval; BMI, body mass index; RHR, resting heart rate.

**Figure 2:**
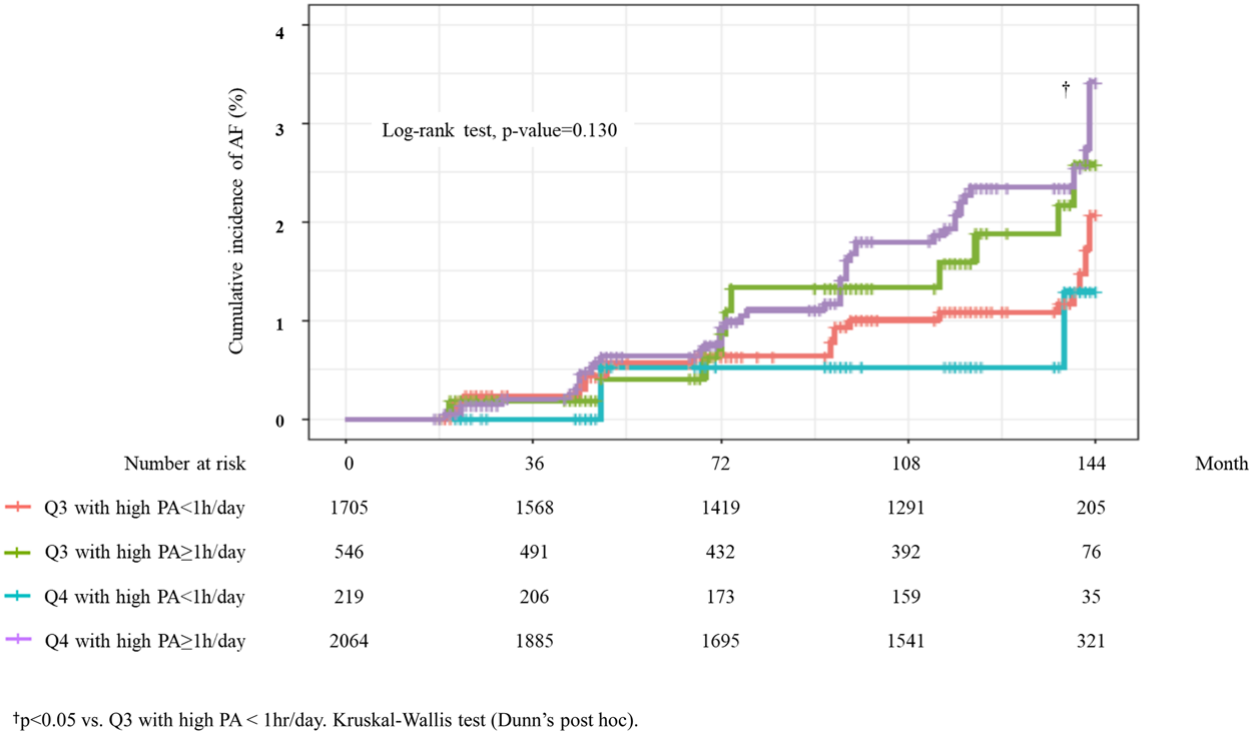
Incidence of atrial fibrillation in relation to presence or absence of high activity (MET 7) ≥ 1 hr/day in Q3 and Q4. ^†^p < 0.05 vs. Q3 with high PA < 1 hr/day. Kruskal-Wallis test (Dunn’s post hoc).

However, when participants in Q3 and Q4 were divided into subgroups, i.e., high PA ≥ 1 hr/day versus high PA < 1 hr/day (Fig. 2), subgroup of Q4 with high PA ≥ 1 hr/day had a significantly higher risk of AF compared with the subgroup of Q3 with high PA < 1 hr/day. Conversely, the risk of AF in the subgroup of Q4 with high PA < 1 hr was not higher than in the subgroup of Q3 with high PA < 1 hr.

Table 4 shows that subjects with RHR < 60 had a higher risk of AF than those with RHR 70–85 bpm. Subjects with RHR 50–59 bpm had a significant risk of AF (HR 1.97, 95% CI 1.17–3.32, p = 0.011). These relationships remained consistent after adding PA to the covariates.

**Table 4:** Hazard ratio according to resting heart rate and incident atrial fibrillation.

		Resting Heart Rate (bpm)				
		<50	50–59	60–70	70–85	>85
No. with AF		5 (3.6%)	52 (2.8%)	87 (1.7%)	21 (1.3%)	2 (2.2%)
Incidence rate		3.74	2.81	1.75	1.40	2.55
Crude	HR (95% CI)	2.69 (1.01–7.12)	2.00 (1.21–3.32)	1.24 (0.77–2.00)	1	1.93 (0.45–8.24)
	p-value	0.047	0.007	0.370		0.374
Model 1	HR (95% CI)	2.54 (0.95–6.79)	1.86 (1.11–3.12)	1.24 (0.77–2.00)	1	1.75 (0.41–7.46)
	p-value	0.064	0.018	0.375		0.452
Model 2	HR (95% CI)	2.54 (0.94–6.88)	1.93 (1.14–3.26)	1.27 (0.79–2.06)	1	1.82 (0.43–7.83)
	p-value	0.066	0.015	0.327		0.419
Model 3	HR (95% CI)	2.56 (0.95–6.88)	1.97 (1.17–3.32)	1.27 (0.79–2.06)	1	1.83 (0.43–7.87)
	p- value	0.062	0.011	0.323		0.415

Model 1: adjusted for age, sex, residence, education.

Model 2: Model 1 + BMI, comorbidity, alcohol, and smoking.

Model 3: Model 2 + physical activities.

AF, atrial fibrillation; Incidence rate, per 1000 person-year; HR, hazard ratio; CI, confidence interval; BMI, body mass index.

## Discussion

Analysis of a prospective cohort based on the general population showed that moderate total PA was associated with a reduced risk of incident AF compared with that for lowest total PA and that the protective effect of PA was attenuated in subjects with highest total PA. Also, low RHR (<60 bpm) was a risk factor for incident AF. These findings remained consistent after adjusting for PA.

Endurance athletes have a lower RHR and are also predisposed to AF^10^. Suggested mechanisms include atrial remodeling, atrial ectopy, and increased vagal tone^11^. The risk of AF increases substantially in the aging athlete with accumulation of lifetime training hours and participation in the competition. However, our study showed that the association between PA and RHR in the general population was not straightforward. There appeared to be some inverse association between PA and RHR up to moderate total PA. However, the highest total PA was not associated with lower RHR. These results suggest that regular PA (even the most vigorous PA) in the general population may be insufficient to affect autonomic tone. Therefore, RHR could not mediate an association between PA and incident AF, similar to the finding in a previous study^3^. Indeed, only 1.6% of participants in our study had RHR < 50 bpm.

Previous meta-analyses found no significant association between PA and AF, or only a non-significant trend^4,12^. However, while these studies only focused on each level of PA to investigate the incidence of AF, various levels and types of PA occur during daily life in the general population, which may have resulted in inconsistent data for the relationship between each level of PA and AF. To that end, we analyzed the relationship between PA and AF using individual total PA levels, which have not been done by others. We quantified daily life PA by using an index based on METs and observed the association between PA and AF in the general population. Interestingly, many in the Q4 group performed some light- and moderate-level PA. However, almost 90% of Q4 subjects performed high-level PA, which attenuated the favorable effect of moderate PA. These findings are consistent with previous studies that showed a higher risk of AF in high- or vigorous-activity^8^ and high level-PA attenuated the benefit of moderate PA^3^. Several pathways have been suggested to explain the mechanism by which PA affects the risk of AF^13^. Moderate PA modulates cardiovascular risk factors and markers of inflammation^14,15^. These positive effects may reduce the risk of AF.

Low RHR was associated with high AF incidence in subjects aged ≥65 years^16^, and low exercise heart rate based on a moderate workload was a long-term predictor of incident AF in middle-aged men^17^. Although the authors used a different range of heart rate as a ref.^16,17^, they consistently suggested that low RHR is associated with high AF. We also found low RHR being associated with a high incidence of AF; these associations were independent of PA. While low RHR was associated with higher incident AF in trained athletes^5^, participants in our study were not trained athletes. Therefore, it is conceivable that mechanisms other than autonomic change, atrial dilation, and fibrosis may play a role. One possible mechanism is subclinical sinus node dysfunction, which may predispose to AF^18^. Increased heart rate was also found to be associated with risk of AF in the Losartan Intervention For Endpoint Reduction in Hypertension (LIFE) study^19^. In the present study, the incident rate of AF and hazard ratio in the ‘RHR > 85 bpm’ group were higher than in the ‘RHR 70–85 bpm’ group but not statistically significant, possibly due to a small number of subjects in this group. A study by Aladin *et al*^20^. reported that the significant association between RHR > 85 bpm and risk of AF disappeared after adjusting for exercise capacity.

The incidence of AF in our study was lower than in previous studies of Western populations^21,22^. For example, Schnabel *et al*. reported an incidence of 7.5/1,000 person-years, based on 50 years of observation in the Framingham cohort^22^. The difference in the incidence may be explained by different populations and the methods to identify AF. The prevalence of AF is reportedly 250–325/100,000 in Northeast Asia, which is the lowest in the world^23^. Also, a recent report based on the Korean population showed a low prevalence and incidence rate of AF (0.67% and 1.5–1.7/1,000 person-years, respectively) in a Korean population, similar to our findings^24^. We used clinic ECGs to identify AF. While this method can increase the sensitivity for AF in the general population, paroxysmal AF would not have been identified, and therefore the prevalence would probably have been underestimated. Indeed, Schnabel *et al*. reported that the incidence of AF based on clinic ECGs was 1.6–3.8/1,000 person-years; this was much lower than the rate based on all available sources and was closer to that in our study^22^. To minimize underestimation of incident AF, we also included the data using self-reported physician-determined diagnosis.

Our research has some limitations. In baseline data collection, we did not distinguish between occupational and leisure time activities when calculating total daily PA. In addition, self-reporting questionnaires may not have accurately reflected the level of PA. While we tried to identify AF using both ECGs and self-reported history of physician-determined diagnosis, we could not obtain participants’ International Classification of Disease (ICD) because ICD was considered protected personal information. Therefore, some paroxysmal AF that waxed and waned between each visit could still have been missed. Information about antihypertensive drugs such as beta blockers was not available for this cohort. However, there were no significant differences in the rate of antihypertensive use according to quartiles.

In conclusion, this prospective cohort study of a Korean general population suggested independent associations between total daily PA and incident AF as well as between RHR and incident AF. A moderate amount of total PA was associated with a lower risk of AF. Higher total PA was not associated with a lower RHR. RHR was also associated with incident AF, showing a U-shaped relationship, independent of the amount of total PA. Further prospective research is warranted to clarify whether the intervention of PA and RHR would be beneficial to prevent AF.

## Supplementary information

Supplementary Figure 1 and 2

## Data Availability

The datasets generated during and/or analyzed during the current study are available from the corresponding author on reasonable request.
