# Supplementary material for: Independent effect of physical activity and resting heart rate on the incidence of atrial fibrillation in the general population
Source: Sci Rep. 2019 Aug 2;9:11228. doi: 10.1038/s41598-019-47748-7 (PMC6677819; doi:10.1038/s41598-019-47748-7)

**Independent effect of physical activity and resting heart rate on the incidence of atrial fibrillation in the general population**

Yeon Woo Choi^1†^, Minsu Park^2†^, Young-Hyo Lim^1†^, Jisun Myung^3^, Byung Sik Kim^1^, Yonggu Lee^4^, Jeong-Hun Shin^4^, Hwan-Cheol Park^4^, Jinho Shin^1^, Chun Ki Kim^5^, and Jin-Kyu Park^1*^

^†^This authors contributed equally to this work.

^1^ Division of Cardiology, Department of Internal Medicine, Hanyang University Medical Center, Seoul, Republic of Korea

^2^ Statistics and Data Center, Samsung Biomedical Research Institute, Samsung Medical Center, Seoul, Republic of Korea

^3^ Department of Preventive Medicine, School of Medicine, Kyunghee University, Seoul, Republic of Korea

^4^ Division of Cardiology, Department of Internal Medicine, Hanyang University Guri Hospital, Guri City, Gyounggi-do, Republic of Korea

^5^ Department of Nuclear Medicine, Hanyang University Medical Center, Seoul, Republic of Korea

***Corresponding Author:**

**Name**: Jin-Kyu Park, M.D., Ph.D.


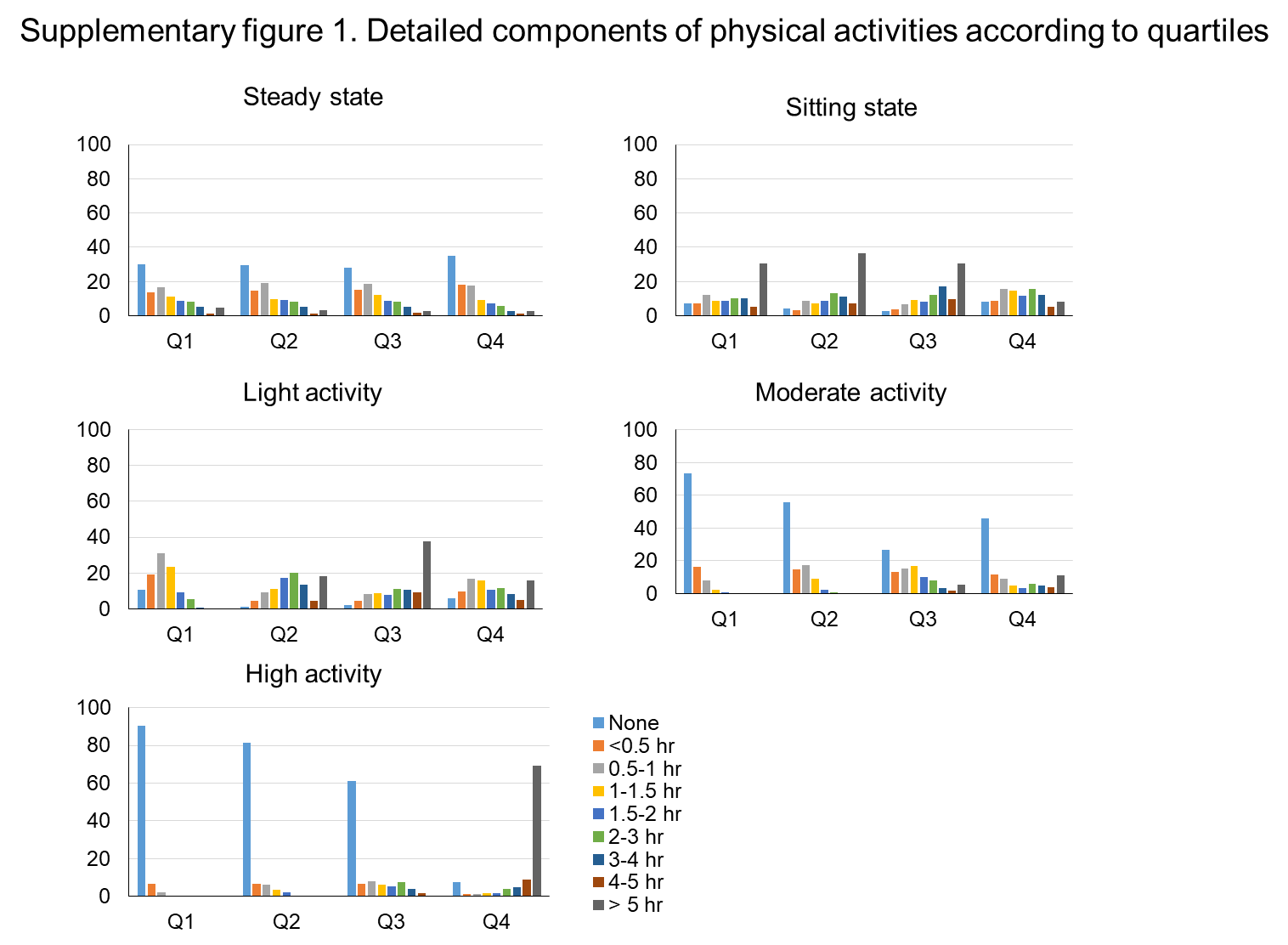


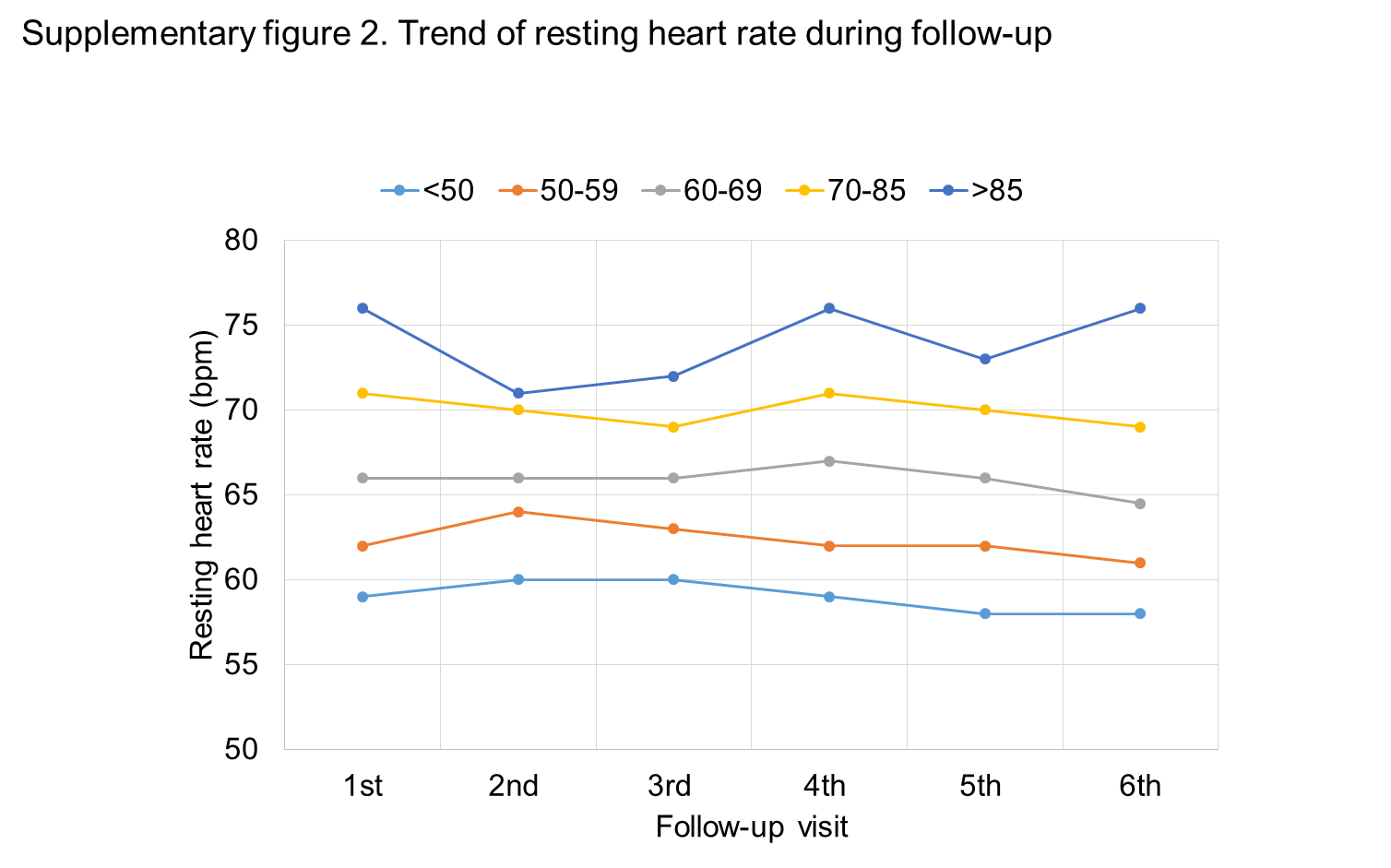

Supplement: Supplementary file 1 — Supplementary Figure 1 and 2 [file 41598_2019_47748_MOESM1_ESM.docx]
